# A New Preparation of Cimicifuga Racemosa

**Published:** 1859-01

**Authors:** 


A NEW PREPARATION OF CIMICIFUGA RACEMOSA.—Dr. John G. Kœhler, of Schuylkill Haven, Pa., has sent us the following communication in regard to this remedy:—

"During the month of July, 1858, I had occasion to use black snakeroot in several cases of chorea; but not finding the desired success with the tincture or decoction, it occurred to me that a more certain preparation could be made. I determined to employ dilute acetic acid and alcohol, and after various experiments as regards strengh, I finally adopted the following formula:—

℞.—Rad. cimicifugæ, ℥v;
Acidi acetici dil. ℥j;
Spts. vini rectif, ℥viij;
Aquæ, ℥xi.

"Digest fourteen days and filter. Dose, ʒj to ʒij.

"After due trial I found this combination to answer better than any other form, and the neighboring physicians, to whom I gave the formula, express themselves as highly pleased with the acetated tincture of cimicifuga. It has been successfully employed in nervous affections, and as an alterative in various forms of rheumatism and uterine affections."

